# Supplementary material for: Plasmonic Metal Nanoparticles in Sensing Applications: From Synthesis to Implementations in Biochemical and Medical Diagnostics
Source: Molecules. 2025 Dec 12;30(24):4745. doi: 10.3390/molecules30244745 (PMC12735965; doi:10.3390/molecules30244745)
Supplement: Supplementary file 1 [file molecules-30-04745-s001.zip › molecules-3999909-supplementary.pdf]

Supplementary Material

Plasmonic Metal Nanoparticles in Sensing Applications: From Synthesis to Implementations in Biochemical and Medical Diagnostics

by Grace Nemeth, Jacob Speers, Salman Shaheen and Vladimir Kitaev

Table S1. Summary of the recent reviews relevant for plasmonic metal nanoparticles (PMNPS) in sensing and relevant closely related fields.

Ref. # (main text)	Year	Authors	Content
(10)	2023	Hanson, E. K.; Whelan, R. J.	Literature review from 2016 to 2022 on the Application of the Nicoya OpenSPR (Surface Plasmon Resonance) to Studies of Biomolecular Binding.
(11)	2025	Cho, S. H.; Choi, S.; Suh, J. M.; Jang, H. W.	Advancements in Surface Plasmon Resonance (SPR) Sensors for Real-Time Detection of chemical analytes, including sensing materials and applications.
(12)	2024	Zorlu, T. et al.	Metal–Organic Frameworks Photocatalyst Through Plasmon-Induced Hot-Electrons (Focus on catalysis and MOFs).
(13)	2025	Yi, J. et al.	Surface-Enhanced Raman Spectroscopy (SERS): A Half-Century Historical Perspective.
(14)	2025	Wang, H.	A Repertoire of Gold-Based Nanostructures with Integrated Optical and Catalytic Tunabilities (Focus on nanostructure tunability).
(15)	2024	Serna-Gallén, P.; Mužina, K.	Metallic Nanoparticles at the Forefront of Research: Novel Trends in Catalysis and Plasmonics.
(16)	2025	Kulakovich, O. et al.	Multifaceted Effects of the Dielectric Component within Plasmon-Assisted Light-Emitting Structures (Focus on optical materials).
(17)	2025	Feng, Y. et al.	Plasmonic Photoelectric Detection Engineering: Basic Principle, Design Strategies and Challenges.
(18)	2023	Babicheva, V. E.	Optical Processes behind Plasmonic Applications (Overview of fundamental processes).
(19)	2025	Zhuang, L.; Lian, Y.; Zhu, T.	Multifunctional Gold Nanoparticles: Bridging Detection, Diagnosis, and Targeted Therapy in Cancer (Focus on biomedicine).
(20)	2023	Zhdanov, V. P.	Basics of the LSPR Sensors for Soft Matter at Interfaces.

(21)	2023	Li, Y.; Liao, Q.; Hou, W.; Qin, L.	Silver-Based Surface Plasmon Sensors: Fabrication and Applications.
(22)	2025	Lin, X. et al.	Recent Advances in Localized Surface Plasmon Resonance (LSPR) Sensing Technologies.
(23)	2022	Hamza, M. E.; Othman, M. A.; Swillam, M. A.	Detailed review on Plasmonic Biosensors.
(24)	2018	Zhang, Z. et al.	Plasmonic Colorimetric Sensors based on etching and growth of noble metal nanoparticles: Strategies and Applications.
(25)	2022	Das, C. M.; Kong, K. V.; Yong, K.-T.	Diagnostic Plasmonic Sensors: Opportunities and Challenges.
(26)	2023	Akgönüllü, S.; Denizli, A.	Plasmonic Nanosensors for Pharmaceutical and Biomedical Analysis.
(27)	2022	Geng, H. et al.	Noble Metal Nanoparticle Biosensors: From Fundamental Studies toward Point-of-Care Diagnostics.
(28)	2023	Wu, Y. et al.	Colorimetric Sensors for Chemical and Biological Sensing Applications.
(29)	2025	Song, H. et al.	Nanoparticle-Integrated Hydrogels as Versatile Colorimetric Sensors (with focus on PMNPs).
(30)	2024	Bhalla, N.; Shen, A. Q.	Localized Surface Plasmon Resonance Sensing and Its Interplay with Fluidics.
(31)	2023	Zhang, W. et al.	Plasmonic Nanomaterials in Dark Field Sensing Systems.
(32)	2024	Kant, K. et al.	Plasmonic Nanoparticle Sensors: Current Progress, Challenges, and Future Prospects.
(33)	2023	Wu, J. Z. et al.	Metallic and Non-Metallic Plasmonic Nanostructures for LSPR Sensors (broader perspective including materials like graphene).
(34)	2023	Zhang, H. et al.	Recent Advancements of LSPR Fiber-Optic Biosensing: Combination Methods, Structure, and Prospects.
(35)	2011	Rycenga, M. et al.	Fundamental Review on Controlling the Synthesis and Assembly of Silver Nanostructures for Plasmonic Applications.
(36)	2017	Reguera, J. et al.	Anisotropic Metal Nanoparticles for Surface Enhanced Raman Scattering (SERS).
(37)	2012	Dykman, L.; Khlebtsov, N.	Gold Nanoparticles in Biomedical Applications: Recent Advances and Perspectives.
(38)	2024	Karnwal, A. et al.	Gold Nanoparticles in Nanobiotechnology: From Synthesis to Biosensing Applications.
